# Let's Have a Chat: How Well Does an Artificial Intelligence Chatbot Answer Clinical Infectious Diseases Pharmacotherapy Questions?

**DOI:** 10.1093/ofid/ofae641

**Published:** 2024-10-25

**Authors:** Wesley D Kufel, Kathleen D Hanrahan, Robert W Seabury, Katie A Parsels, Jason C Gallagher, Conan MacDougall, Elizabeth W Covington, Elias B Chahine, Rachel S Britt, Jeffrey M Steele

**Affiliations:** School of Pharmacy and Pharmaceutical Sciences, Binghamton University, Binghamton, New York, USA; State University of New York Upstate University Hospital, Syracuse, New York, USA; State University of New York Upstate Medical University, Syracuse, New York, USA; State University of New York Upstate University Hospital, Syracuse, New York, USA; State University of New York Upstate University Hospital, Syracuse, New York, USA; State University of New York Upstate Medical University, Syracuse, New York, USA; State University of New York Upstate University Hospital, Syracuse, New York, USA; School of Pharmacy, Temple University, Philadelphia, Pennsylvania, USA; School of Pharmacy, University of California San Francisco, San Francisco, California, USA; Harrison College of Pharmacy, Auburn University, Auburn, Alabama, USA; School of Pharmacy, Palm Beach Atlantic University Gregory, West Palm Beach, Florida, USA; University of Texas Medical Branch at Galveston, Galveston, Texas, USA; State University of New York Upstate University Hospital, Syracuse, New York, USA; State University of New York Upstate Medical University, Syracuse, New York, USA

**Keywords:** artificial intelligence, chatbot, ChatGPT, infectious diseases, pharmacist

## Abstract

**Background:**

It is unknown whether ChatGPT provides quality responses to infectious diseases (ID) pharmacotherapy questions. This study surveyed ID pharmacist subject matter experts (SMEs) to assess the quality of ChatGPT version 3.5 (GPT-3.5) responses.

**Methods:**

The primary outcome was the percentage of GPT-3.5 responses considered useful by SME rating. Secondary outcomes were SMEs' ratings of correctness, completeness, and safety. Rating definitions were based on literature review. One hundred ID pharmacotherapy questions were entered into GPT-3.5 without custom instructions or additional prompts, and responses were recorded. A 0–10 rating scale for correctness, completeness, and safety was developed and validated for interrater reliability. Continuous and categorical variables were assessed for interrater reliability via average measures intraclass correlation coefficient and Fleiss multirater kappa, respectively. SMEs' responses were compared by the Kruskal-Wallis test and chi-square test for continuous and categorical variables.

**Results:**

SMEs considered 41.8% of responses useful. Median (IQR) ratings for correctness, completeness, and safety were 7 (4–9), 5 (3–8), and 8 (4–10), respectively. The Fleiss multirater kappa for usefulness was 0.379 (95% CI, .317–.441) indicating fair agreement, and intraclass correlation coefficients were 0.820 (95% CI, .758–.870), 0.745 (95% CI, .656–.816), and 0.833 (95% CI, .775–.880) for correctness, completeness, and safety, indicating at least substantial agreement. No significant difference was observed among SME responses for percentage of responses considered useful.

**Conclusions:**

Fewer than 50% of GPT-3.5 responses were considered useful by SMEs. Responses were mostly considered correct and safe but were often incomplete, suggesting that GPT-3.5 responses may not replace an ID pharmacist's responses.

## Teaser

Infectious diseases (ID) pharmacists' assessment of ChatGPT version 3.5 responses to ID pharmacotherapy questions indicated that <50% of responses were considered useful, while most were considered correct and safe but were often incomplete, suggesting that ChatGPT responses may not replace ID pharmacists' responses.

Large language models (LLMs) are artificial intelligence (AI) tools with extensive learning algorithms that provide the ability to comprehend and generate extensive amounts of text data [1, 2]. One such LLM is Chat-Generative Pre-trained Transformer (ChatGPT), a conversational open-access chatbot with >175 billion parameters [3]. ChatGPT's training data encompass a broad range of internet sources, including books, articles, and websites. LLMs have been explored in the health care setting in various capacities, such as their ability to navigate clinical vignettes and answer clinical questions [4–6]. One study demonstrated that ChatGPT performed at or near the passing threshold for all 3 United States Medical Licensing Examinations, with a high level of concordance and insight in its explanations as compared with 2 physician adjudicators [7]. Whether performance on these examinations translates to providing correct and meaningful responses to real-life complex medical questions is unknown, especially given concerns about the factual inaccuracies of LLMs [8–11].

The field of infectious diseases (ID) is becoming increasingly complex and has seen a decline in the number of physicians pursuing careers in it [12–14]. This is juxtaposed with a mandate by the Joint Commission for Acute Care Hospitals to have antimicrobial stewardship programs, which are often co-led or led by an ID pharmacist [15–17]. The push for institutions to have such programs has resulted in the majority of US hospitals having at least 1 pharmacist responsible for antimicrobial stewardship [18]. The level of training required to serve in this role is not standardized, with only 21.1% completing a postgraduate year 2 ID residency and/or ID fellowship and approximately half completing a training course on antimicrobial stewardship. Pharmacists can, however, demonstrate proficiency in ID pharmacotherapy by becoming credentialed as a Board-Certified Infectious Diseases Pharmacist (BCIDP) by the Board of Pharmacy Specialties [19]. Approximately 2000 pharmacists currently hold this designation [19]. ID pharmacists have expertise in antimicrobial pharmacology, clinical microbiology, pharmacokinetics, and pharmacodynamics and are frequently familiar with current ID literature [20, 21]. Clinicians, including ID physicians, commonly seek out ID pharmacists for their expertise to answer clinical ID pharmacotherapy questions pertaining to antimicrobials [21]. There is also a growing need for ID pharmacists in outpatient antimicrobial stewardship programs [17]. Yet, ID pharmacists may not be readily available at all institutions or at all hours of the day; therefore, providers may rely on AI software tools such ChatGPT to answer clinical ID pharmacotherapy questions [22]. Given the potential for LLMs to generate potentially useful responses, we sought to evaluate how well ChatGPT version 3.5 (GPT-3.5), a free-to-use AI chatbot, answered ID pharmacotherapy questions from the perspective of ID pharmacists.

## METHODS

### Study Outcomes

The primary outcome was to assess if GPT-3.5 responses to ID pharmacotherapy questions were useful based on the opinion of ID pharmacist subject matter experts (SMEs). Secondary outcomes were to evaluate the correctness, completeness, and safety of GPT-3.5 responses based on SME opinion.

### Study Design

This cross-sectional survey was distributed to 5 ID pharmacist SMEs. SMEs were required to be practicing ID pharmacists in the United States and meet at least 1 of the following 2 criteria: maintain BCIDP credentialing or have >10 years of clinical ID pharmacy practice experience. Selection of the SMEs was intended to incorporate diversity, equity, and inclusion considerations. The investigators emphasized that participation was voluntary and that data would be kept confidential and used only for research purposes.

### Question Creation

One hundred questions were developed by 2 BCIDP investigators and were modeled after the types of clinical ID pharmacotherapy questions asked by providers, including ID physicians, to ID pharmacists in the inpatient setting (Supplementary Appendix 1). The investigators opted for 100 questions to represent a diverse bank of clinical ID pharmacotherapy questions. Questions were developed in the following categories: drug selection, drug dosing, drug interactions, adverse effects/drug monitoring, therapeutic drug monitoring, antimicrobial prophylaxis, antimicrobial resistance, and clinical microbiology. Sample questions based on category can be found in Supplementary Appendix 2. Individual questions within the question bank were rated according to difficulty by the 2 investigators who developed the questions, and a third BCIDP investigator was asked to independently rate questions when discordance existed in difficulty rating. Question difficulty was defined as follows: *easy*, information is readily retrievable via clinical practice guidelines and/or secondary or tertiary references; *medium*, some assessment is required in conjunction with information that is readily retrievable from guidelines and/or secondary or tertiary references; and *hard*, information is not available in guidelines, requires interpretation of primary literature, and/or represents a clinical situation for which there is not a standard of care. Examples of questions based on question difficulty can be found in Supplementary Appendix 3.

### Generation of Chatbot Responses and Study Instrument Development

Questions were consecutively entered into GPT-3.5 by a single prompt, and each question was asked as a new conversation to eliminate possible learning from previous answers. Responses were recorded into a Microsoft Excel spreadsheet. GPT-4o or GPT-4 was not freely available at the time of this study; as such, GPT-3.5 was used.

Definitions were developed for *correct*, *complete*, *safe*, and *useful*: *correct*, the response contains true information, even if it is incomplete or vague; *complete*, the response includes all relevant information necessary to answer the question even if there is inaccurate information; *safe*, action or inaction based on the response does not have the potential to cause danger, risk, or injury; *useful*, the response provides the information necessary to adequately answer the question without the need for additional resources. A 0–10 rating scale was also developed to assess the correctness, completeness, and safety of chatbot responses. The scale was labeled at the poles and midpoint. For correctness, 10 is “entirely correct,” 5 is “equal parts of correct and incorrect,” and 0 is “entirely incorrect.” For completeness, 10 is “all parts of the response are provided to sufficiently answer the question,” 5 is “half the parts are provided to sufficiently answer the question,” and 0 is “none of the parts are provided to sufficiently answer the question.” For safety, 10 is “no danger, risk, or injury,” 5 is “moderate danger, risk, or injury,” and 0 is “significant danger, risk, or injury.”

The 2 BCIDP investigators who developed the question bank selected a random sampling of 15 questions and the associated GPT-3.5 responses to test the rating scales and definitions for correctness, completeness, safety, and usefulness. The rating scales and definitions for the ordinal categories were adjusted until the investigator responses achieved an intraclass correlation coefficient (ICC) >0.60, indicating substantial agreement. The rating scale and definitions for the categorical category were adjusted until the investigator responses achieved a kappa statistic >0.60, indicating substantial agreement.

### Survey

The 100 questions, associated GPT-3.5 responses, developed definitions, and rating scales were entered into REDCap as a survey instrument [23, 24]. Demographic questions were also incorporated into the survey instrument to collect gender, race and ethnicity, years in practice, board certification/degrees, and practice setting. The REDCap survey was distributed via email to 5 SMEs. SMEs were asked to review each question and rate the associated GPT-3.5 response using the provided scale for correctness, completeness, and safety and to rate the response as useful or not. Prior to starting the survey, an investigator met with the SMEs individually to explain the evaluation process, definitions, and rating scales as well as to answer any questions to promote understanding. The survey instrument was able to be saved and resumed later to minimize evaluation fatigue.

### Statistical Analysis

Statistical analyses were conducted with SPSS version 29.0 (IBM). All data were presented by descriptive statistics, including number with percentage and median with IQR. Interrater reliability was assessed for ordinal variables via an average measures ICC. ICC results were presented with 95% CIs. Interrater reliability was assessed for categorical variables with Fleiss multirater kappa and presented with a 95% CI. Interrater agreement was assessed with the following scale for kappa and ICC results: ≤0.20, poor to slight agreement; 0.21–0.40, fair agreement; 0.41–0.60, moderate agreement; 0.61–0.80, substantial agreement; 0.81–0.99, almost perfect agreement; and 1.0, perfect agreement [25]. Categorical and ordinal variables were compared across groups with the chi-square test for independence and the Kruskal-Wallis test. All tests were 2-tailed, and a *P* < .05 was considered statistically significant. A post hoc analysis was performed by the Mann-Whitney *U* test to identify the location of the significant difference found in the safety variable based on question difficulty (easy, medium, hard). This test was again 2-tailed, and a *P* < .017 was considered statistically significant after Bonferroni correction. An additional post hoc analysis was performed to assess if GPT-3.5 recommended consultation at different frequencies among easy, medium, and hard questions. This analysis was performed via a 3 × 2 chi-square test and was 2-tailed, with a *P* value of .05 considered statistically significant.

## RESULTS

The demographics of the 5 SMEs are displayed in Supplementary Table 1. Overall, there was diverse representation of SMEs based on gender, race and ethnicity, years in practice, primary clinical practice site, and geographic location. GPT-3.5 response ratings by the SMEs are displayed in Table 1. For the primary outcome, the SMEs determined that the GPT-3.5 responses were useful 41.8% (209/500) of the time. For secondary outcomes, SMEs rated correctness, completeness, and safety at medians of 8 (IQR, 4–9), 5 (IQR, 3–8), and 8 (IQR, 4–10), respectively.

**Table 1.** Primary and Secondary Outcome Results (n = 500)

Outcome	SME Responses
Primary, No. (%)	
Useful	209 (41.8)
Secondary, median (IQR)	
Correctness	7 (4–9)
Completeness	5 (3–8)
Safety	8 (4–10)

Abbreviation: SME, subject matter expert.

The interrater agreements among SMEs are described in Table 2. The ICCs for usefulness, correctness, completeness, and safety were 0.379 (95% CI, .317–.441), 0.82 (95% CI, .758–.870), 0.745 (95% CI, .656–.816), and 0.833 (95% CI, .775–.880), respectively. Interpretations of the interrater agreements for useful, correctness, completeness, and safety were fair, almost perfect, substantial, and almost perfect, respectively. Therefore, response ratings among SMEs were in agreement for the majority of rating categories.

**Table 2.** Interrater Agreements Among Subject Matter Experts (n = 500)

	Results (95% CI)	Agreement
Fleiss multirater kappa		
Useful	0.379 (.317–.441)	Fair
Average measures ICC		
Correctness	0.820 (.758–.870)	Almost perfect
Completeness	0.745 (.656–.816)	Substantial
Safety	0.833 (.775–.880)	Almost perfect

Abbreviation: ICC, intraclass correlation coefficient.

Table 3 describes the correctness, completeness, safety, and usefulness of the SME responses based on question difficulty and category. There were no statistically significant differences in correctness, completeness, or useful based on question difficulty. However, there was a statistically significant difference in safety based on question difficulty, with the easy questions having higher safety ratings as compared with the medium and hard questions. There were no statistically significant differences in safety ratings with medium questions vs hard questions. There were no statistically significant differences in usefulness, correctness, completeness, or safety for any of question categories.

**Table 3.** Evaluation of Question Difficulty and Question Category

Question Difficulty	Easy (n = 280)	Medium (n = 160)	Hard (n = 60)	*P* Value
Useful	128 (45.7)	60 (37.5)	21 (35)	.127
Correctness	8 (4–9)	7 (4–9)	7 (5–9)	.123
Completeness	5 (3–8)	5 (3–8)	5 (3–7.8)	.433
Safety	8 (5–10)^a^	7 (3–9)^a^	7 (4.3–9)	.008

Question Category	Drug Selection or Dosing (n = 275)	Drug Interactions, Adverse Effects, or Monitoring (n = 165)	Antimicrobial Resistance or Microbiology (n = 60)	
Useful	114 (41.5)	72 (43.6)	23 (38.3)	.764
Correctness	8 (4–10)	7 (4–9)	7 (5–9)	.271
Completeness	5 (3–8)	5 (3–8)	4.5 (2–7.8)	.211
Safety	8 (3–10)	8 (3–10)	8 (5–9.8)	.566

Data are presented as No. (%) or median (IQR).

^a^Statistical significance at .017 by Bonferroni correction.

For 67 responses among the 100 questions, GPT-3.5 recommended consulting an ID specialist, pharmacist, and/or health care provider. There was no statistically significant difference in the frequencies in which GPT-3.5 recommended consultation among easy, medium, or hard questions (62.5% vs 71.9% vs 75.0%, *P* = .548).

## DISCUSSION

This study assessed how well GPT-3.5 answered clinical ID pharmacotherapy questions from the perspective of ID pharmacists. For the primary outcome, 41.8% of responses were considered useful, although the level of agreement among raters for useful was considered fair, which may lower the confidence in this result. SMEs identified that the responses largely provided information that was correct (median, 7; IQR, 4–9) and safe (median, 8; IQR, 4–10), but responses often were incomplete (median, 5; IQR, 3–8).

Some questions were written for a potential *yes/no* response, but GPT-3.5 frequently gave more than a *yes* or *no* response to substantiate its answer and, in doing so, provided incorrect information or an incomplete rational to support the answer. Unlike the responses provided by GPT-3.5, ID pharmacists provide answers to queries that are (1) qualified by the available level of evidence, (2) nuanced with associated clinical reasoning based on the specific situation, and (3) supported by evidence-based literature [21]. Out of 100 GPT-3.5 responses, 67 recommended consulting an ID specialist, pharmacist, and/or health care provider. There was no significant difference in the difficulty of the question (ie, easy, medium, hard) for when GPT-3.5 recommended consultation. When examining ID pharmacist SME ratings based on question category, we identified no differences. However, GPT-3.5 responses to medium and hard questions were more likely to return unsafe recommendations as compared with responses to easy questions (*P* = .008). GPT-3.5 did not perform better on easier questions, as might be expected for correctness, completeness, or useful. The general absence of an association with question type or difficulty may be illustrative of the unpredictable nature of LLMs, the risk of LLM hallucinations, and the “jagged frontier” representing the potential boundaries of the performance of these tools; yet, it may also be affected by the type of question or the potential for responses to be associated with some degree of clinical controversy [26]. Considering these findings, GPT-3.5 may not be in a position to usurp ID pharmacists, who may be more capable of providing well-formulated, evidence-based responses to clinical ID pharmacotherapy questions.

Goodman et al evaluated answers generated by GPT-3.5 and GPT-4 to 180 questions provided by 33 physicians from 17 specialties approximately 3 months after their introduction [4]. They used a 6-point Likert scale for accuracy and a 3-point Likert scale for correctness and completeness. The median accuracy and completeness scores were 5 (mean, 4.4; SD, 1.7) and 3 (mean, 2.4; SD, 0.7), respectively. Scores were also assessed by question difficulty and question type (binary vs descriptive), and no differences were found. Although we used a 0–10 scale as opposed to a 3- or 6-point scale and used the term *correctness* rather than *accuracy*, the results from our study appear similar, but scores for completeness were higher in the study conducted by Goodman et al. The differences in ratings may be attributed to the authors being asked to formulate questions with clear and uncontroversial answers from available medical guidelines, whereas we developed questions that were designed to involve clinical reasoning. We also used a 0–10 rating scale that offers more granularity in responses, which may have resulted in differences in scores.

Beavers et al evaluated responses from pharmacists, an AI chatbot (Micromedex with Watson), and a traditional medication database to 200 real-world medication use questions [6]. Pharmacists' responses were considered the reference response. A group of pharmacists, nurses, and providers rated the responses for correctness, completeness, safety, and usefulness using a 9-point scale. Pharmacists' mean (SD) scores for responses provided by the chatbot for correctness, completeness, safety, and usefulness were 7.1 (2.60), 5.77 (3.12), 6.35 (2.70), and 5.63 (2.94), respectively. All ratings were significantly lower for the chatbot responses as compared with the reference responses. Nurse and provider ratings of chatbot responses were also significantly lower than ratings of pharmacists' responses. The authors considered chatbot responses acceptable if the mean SME rating was within 3 points of the reference answer. Using this definition, they found that 88%, 76%, 83%, and 81% of responses were acceptable for correctness, completeness, safety, and usefulness based on pharmacists' ratings. In our study, 41.8% of responses were considered useful. We used a binary assessment (yes or no) to determine if a response was useful or not, which may have contributed to the lower percentage of responses considered useful in our study. Similar to our study, responses for completeness were low, which may have affected usefulness scores. Rating differences may also be attributed to the different chatbots used.

Limited data are available to describe the ability of ChatGPT to answer real-world ID questions [9, 27–29]. Howard et al provided a narrative account of the performance of GPT-3.5 in providing antimicrobial advice in 8 ID scenario-based hypothetical questions [29]. Antimicrobial spectra and regimens were appropriate for the diagnosis and recognized the implications of clinical response. Appropriateness for duration of therapy varied, especially when source control was a factor. GPT-3.5 was often not able to differentiate the level of importance when a contraindication was identified for an antimicrobial, sometimes ignored its own advice, and facilitated inappropriate management plans. The authors also noted that most responses included disclaimers that recommended consultation with an infection specialist, as described in our study. They concluded that the largest barriers to the implementation of ChatGPT in clinical practice are deficits in situational awareness, inference, and consistency. Sarink et al evaluated GPT-3.5 responses among actual ID cases and compared these responses with the advice provided by consultants [27]. They found that correct advice was often supported by misrepresented supporting evidence, and they suggested that the responses may even put patients at risk. In many of these aforementioned studies and in real-world settings, clinicians are unlikely using techniques to optimize their search result, which could affect the ChatGPT response [27–30]. Future studies should evaluate if prompting a ChatGPT in a certain manner or using custom instructions will optimize search results for clinical ID questions and increase their usefulness.

Our study has several strengths. First, definitions and rating scales were internally validated prior to sending responses to SMEs, and SMEs were individually instructed on how to use the rating scales. Nearly perfect agreement among SMEs was observed for correctness and safety, and at least fair agreement was seen for all categories. We also queried GPT-3.5 with a single prompt and used a new conversation to avoid responses being skewed by previous questions. Unlike other studies, we used a 10-point rating scale, which allowed more discrimination in responses. Furthermore, most of the questions developed were designed to involve clinical reasoning and represent real-world ID pharmacotherapy questions as compared with questions that had direct answers. We also attempted to integrate diversity, equity, and inclusion in the selection of SMEs.

Nonetheless, this study is not without limitations. Since questions were developed as real-world examples, some required clinical reasoning that lacked an unequivocal answer or may have been associated with some degree of clinical controversy, which may have added additional subjectivity to the SMEs' ratings. Techniques such as multishot prompting, prompt engineering, specialized prompt wording, or use of the custom instructions feature may improve the performance of LLMs on clinical questions but are not likely to represent how clinicians would interact with a chatbot; different prompts can have variable effects across various LLMs as well [31]. There may have been a natural tendency among the SMEs to score the responses more harshly, because they do not want a chatbot potentially “replacing” their ID pharmacist role. Our study lacked an ID pharmacist comparator group, but future studies should consider blinding survey respondents to answers provided by ID pharmacists vs those by LLMs to minimize this potential bias. Our results also reflect the performance of GPT-3.5 at the single time queried, and responses may have varied if questions were entered into more recent versions of ChatGPT or other types of chatbots or at different periods, since the performance of LLMs is expected to change as models continue to learn. Although GPT-4 has demonstrated improved performance in biomedical knowledge domains and reductions in hallucinations as compared with GPT-3.5 [32–34], this version was not freely available at the time of this study, nor was GPT-4o. Furthermore, the versions of ChatGPT continue to be updated at a rapid pace, making evaluation of the most current version logistically challenging.

## CONCLUSION

Based on the findings of this study, it is evident that while GPT-3.5 can provide responses to clinical ID pharmacotherapy questions that are mostly correct and safe, its utility is limited by the frequent incompleteness of its answers. GPT-3.5's performance, as evaluated by ID pharmacists, suggests that it cannot yet replace the nuanced, evidence-based, and contextually rich responses by ID pharmacists. Therefore, while GPT-3.5 may serve as a supplementary tool, it should not be relied on as a primary source for clinical decision making in ID pharmacotherapy. Future advancements in AI technology and further research into optimizing AI prompting could enhance its usefulness, but for now, the expertise of trained ID pharmacists remains indispensable.

## Supplementary Material

ofae641_Supplementary_Data

## Supplementary Data

Supplementary materials are available at *Open Forum Infectious Diseases* online. Consisting of data provided by the authors to benefit the reader, the posted materials are not copyedited and are the sole responsibility of the authors, so questions or comments should be addressed to the corresponding author.

## Notes

***Patient consent statement*.** This study was deemed exempt by the Institutional Review Board at the State University of New York Upstate Medical University.

***Prior presentations*.** This study was presented as an oral abstract on 19 October 2024 at IDWeek 2024, Los Angeles, California.

***Financial support*.** This research project was supported by an award by the New York State Council of Health-system Pharmacist Research and Education Foundation.
