# Correction to: Let's Have a Chat: How Well Does an Artificial Intelligence Chatbot Answer Clinical Infectious Diseases Pharmacotherapy Questions?

**DOI:** 10.1093/ofid/ofaf162

**Published:** 2025-03-18

This is a correction to: Wesley D Kufel, Kathleen D Hanrahan, Robert W Seabury, Katie A Parsels, Jason C Gallagher, Conan MacDougall, Elizabeth W Covington, Elias B Chahine, Rachel S Britt, Jeffrey M Steele, Let's Have a Chat: How Well Does an Artificial Intelligence Chatbot Answer Clinical Infectious Diseases Pharmacotherapy Questions?, *Open Forum Infectious Diseases*, Volume 11, Issue 11, November 2024, ofae641, https://doi.org/10.1093/ofid/ofae641

The following changes have been made to the originally published manuscript. A **Funding** section has been added with the statement: “This research project was supported by an award by the New York State Council of Health-system Pharmacist Research and Education Foundation”.

