# Supplementary material for: Let's Have a Chat: How Well Does an Artificial Intelligence Chatbot Answer Clinical Infectious Diseases Pharmacotherapy Questions?
Source: Open Forum Infect Dis. 2024 Oct 25;11(11):ofae641. doi: 10.1093/ofid/ofae641 (PMC11551448; doi:10.1093/ofid/ofae641)
Supplement: ofae641_Supplementary_Data [file ofae641_supplementary_data.docx]

**Supplementary Appendix 1.** Clinical infectious diseases pharmacotherapy questions evaluated.

Drug selection

1. Is cefazolin preferred over vancomycin to treat methicillin-susceptible *Staphylococcus aureus* bacteremia in a patient with anaphylaxis to penicillin?
2. What risk factors should a patient have to receive empiric treatment with vancomycin for community-acquired pneumonia?
3. Should dexamethasone be used empirically in all adult patients with suspected bacterial meningitis?
4. Which antibiotic(s) should be used for *Listeria monocytogenes* coverage in a patient with anaphylaxis to penicillin?
5. What is the clinical efficacy of molnupravir compared to nirmatrelvir/ritonavir for COVID-19?
6. Should micafungin be used for treatment of *Candida albicans* endophthalmitis?
7. Which single tablet regimens are available to treat newly diagnosed HIV?
8. A patient living with HIV is virologically suppressed becomes pregnant while on bictegravir/tenoforvir alafenamide/emtricitabine. Should the regimen be changed?
9. Does bezlotoxumab reduce the risk of *Clostridioides difficile* recurrence if given after antibiotic therapy completion for *Clostridioides difficile*?
10. A patient is identified as having a *Clostridioides difficile* infection with a non-NAP1/01/027 strain. Should vancomycin or fidaxomicin be given to reduce the risk of recurrence?
11. What is the preferred antimalarial to treat severe malaria in a patient traveling from Sudan?
12. What latent tuberculosis treatment should be used in pregnant women for 3 months?
13. How should a pregnant patient with syphilis and a severe IgE-mediated allergy to penicillin be managed?
14. What antibiotics should be used with penicillins to reduce toxin production in toxic shock syndrome caused by *Streptococcus pyogenes?*
15. When should remdesivir be given to an asymptomatic hospitalized patient who tested positive for COVID-19?
16. Is cefepime a viable treatment option for extended-spectrum beta-lactamase producing Enterobacterales bacteremia?
17. A patient with a severe egg allergy was advised to receive their annual influenza vaccine. What influenza vaccine(s) are safe to administer?
18. A patient was bit by a dog with unknown rabies vaccine history. What is the recommended rabies vaccine series for this patient?
19. What treatment options are available to treat trichomoniasis for a patient with a severe IgE-mediated allergy to metronidazole?
20. When should vancomycin be considered as empiric therapy among hospitalized patients with neutropenic fever?
21. A patient is found to be coinfected with both Lyme disease and anaplasmosis. What antibiotic should be used to treat both pathogens?
22. Should daptomycin be used for methicillin-resistant *Staphylococcus aureus* bacteremia and tricuspid valve infective endocarditis with septic pulmonary emboli?
23. What is the recommended vaccine schedule after a patient undergoes splenectomy?
24. When should maribavir be considered to treat cytomegalovirus disease?
25. Which antifungal(s) should be considered to treat a patient with high risk for Mucorales infection?
26. Which combination treatment regimens should be used to treat persistent methicillin-resistant *Staphylococcus aureus* bacteremia?
27. Should cefazolin be used for a methicillin-susceptible *Staphylococcus aureus* epidural abscess?
28. What treatment options are available for *Klebsiella pneumoniae* carbapenemase-producing Enterobacterales pneumonia?
29. What treatment options are available for New Delhi metallo-beta-lactamase-producing Enterobacterales infections?
30. What treatment options are available for carbapenem-resistant *Acinetobacter baumannii* infections?
31. What treatment options are available for trimethoprim-sulfamethoxazole resistant and levofloxacin resistant *Stenotrophomonas maltophilia* pneumonia?
32. Should oral linezolid be used for uncomplicated *Staphylococcus aureus* bacteremia?
33. For which patient populations should letermovir be used for cytomegalovirus prophylaxis?
34. Should additional antiviral prophylaxis be added to letermovir when used for cytomegalovirus prophylaxis in transplant recipients?
35. What is the preferred antifungal for *Candida auris* fungemia?
36. What antimicrobial should be used for primary prophylaxis of *Pneumocystis jirovecii* in a patient living with HIV with a CD4 count of 83 and a sulfa allergy?
37. Should ertapenem be used in a critically ill patient with extended-spectrum beta-lactamase producing Enterobacterales bacteremia with an albumin of 2.0 g/dL?

Drug dosing

1. An 85-year-old male weighing 74 kg with a height of 178 cm and a serum creatinine of 1.1 mg/dL requires vancomycin. What is an appropriate dosing regimen?
2. A patient on thrice weekly hemodialysis has cystitis and requires oral levofloxacin. What is an appropriate dose?
3. What dose of meropenem should be used to treat extended spectrum beta-lactamase-producing Enterobacterales bacteremia in a critically ill patient in the intensive care unit with a creatine clearance > 50 ml/min?
4. A patient living with HIV was scheduled to receive their cabotegravir-rilpivirine dose, but missed their original appointment and now presents 4 days later. Should the patient still receive their dose without any treatment adjustments?
5. How should the dose of liposomal amphotericin be adjusted in patients with a creatinine clearance of 15 ml/min?
6. Calculate a dosing weight for tobramycin for a *Pseudomonas aeruginosa* urinary tract infection in a male patient who weighs 125 kilograms and is 5’7” tall.
7. What dalbavancin dosing regimen should be used to treat acute osteomyelitis?
8. What dose of amoxicillin should be prescribed to a 4-year-old who weighs 20 kg with community-acquired pneumonia?
9. What dose of fluconazole should be used for a bloodstream infection caused by susceptible-dose dependent *Candida glabrata*?
10. Should bictegravir/tenoforvir alafenamide/emtricitabine be used in patients with end stage renal disease receiving intermittent hemodialysis thrice weekly?
11. What baloxavir dose should a 100-kilogram patient with influenza receive?
12. What dose of cefazolin should be used for surgical site infection prophylaxis in a patient weighing 140 kg?

Drug interactions

1. Does increasing the dose of valproic acid overcome the reduction in serum concentration caused by co-administration with a carbapenem?
2. How should the dose of tacrolimus be adjusted when coadministered with isavuconazole?
3. Should ciprofloxacin suspension be administered via an enteral feeding tube?
4. Should tizanidine be held when a patient starts ciprofloxacin?
5. Is linezolid contraindicated with selective serotonin reuptake inhibitors?
6. If a patient is on tube feeds via an enteral feeding tube while also on dolutegravir, what considerations must be taken to avoid a potential drug interaction?
7. Is it necessary to hold simvastatin 80 mg when a patient is receiving daptomycin?
8. What anticoagulant is likely to be the safest for deep venous thrombosis prevention in a patient receiving rifampin?
9. What nucleoside reverse transcriptase inhibitor should be avoided when coadministered with rifampin?
10. Should bictegravir/tenoforvir alafenamide/emtricitabine be crushed and administered via an enteral feeding tube?

Adverse effects/Drug Monitoring

1. Is back pain an adverse effect with intravenous liposomal amphotericin B?
2. Is levofloxacin or ciprofloxacin more likely to prolong the QTc interval?
3. What are risk factors for ethambutol-related toxic optic neuropathy?
4. What risk factors are associated with cefepime-associated neurotoxicity?
5. Is there a risk for hypersensitivity to fidaxomicin if patients have a macrolide allergy?
6. How should leukopenia from valganciclovir be managed?
7. Are patients likely to develop a disulfiram-like reaction if they take metronidazole with alcohol?
8. What effects on the skin does clofazimine have?
9. How should crystalluria-associated nephropathy be mitigated when using intravenous acyclovir?
10. For which antimicrobials should G6PD testing be done prior to starting?
11. Should doxycycline be administered to a 6-year-old patient for 10 days for the treatment of ehrlichiosis?
12. What monitoring should be performed for a patient receiving foscarnet?
13. What beta-lactams share a similar R-1 side chain with cephalexin?
14. How should a patient receiving daptomycin for three weeks be managed if they have a CK level of 1800 u/L without any myalgias?

Therapeutic drug monitoring

1. What is the itraconazole serum concentration target when treating histoplasmosis?
2. Is there a vancomycin trough that is correlated with an area under the concentration-time curve of 400 mg*hr/L in pediatric patients?
3. Is there a cefepime serum concentration that is associated with neurotoxicity?
4. When should therapeutic drug monitoring of antimycobacterial agents be considered?
5. What is the target posaconazole serum concentration when used to prevent invasive fungal infections?
6. How should the dose of voriconazole be adjusted if a 200 mg by mouth twice daily dosing regimen resulted in a serum trough concentration of 5.7 mg/L?
7. Calculate the AUC_24_ for a patient receiving vancomycin 1 gram every 12 hours infused over 1 hour with a peak concentration of 34.5 mg/L two hours after the end of the 4th dose and a trough concentration of 13.1 mg/L, 30 minutes before the 5th dose.
8. Is there a greater risk for nephrotoxicity when the vancomycin AUC24 is greater than 600 mg*hr/L?
9. What is the AUC24 estimation for a patient with a serum level of 25 mcg/mL receiving vancomycin 2500 mg as a continuous infusion daily?

Antimicrobial prophylaxis

1. Should cefazolin be used for surgical prophylaxis in an adult patient undergoing total knee arthroscopy if the patient experiences hives to penicillin as a child?
2. Is primary prophylaxis needed for *Mycobacterium avium complex* in a patient living with HIV who started highly active antiretroviral therapy?
3. Should dapsone be used as primary prophylaxis for *Toxoplasma gondii* in a patient living with HIV with a CD4 count of 75?
4. Should ethanol locks be used to prevent catheter-related bloodstream infections?
5. Should doxycycline 200 mg as a single dose be used for post-exposure prophylaxis following possible exposure to sexually transmitted infections?
6. What are the criteria for doxycycline post-exposure prophylaxis following a tick bite from the *Ixodes scapularis*?

Antimicrobial resistance

1. Should amphotericin B be used to treat all species of *Candida*?
2. Should flucytosine be used to treat a *Candida krusei* urinary tract infection?
3. What is the clinical implication of the M184V gene for antiretroviral therapy in a patient living with HIV?
4. Which bacteria are associated with clinically relevant inducible AmpC production?
5. Can ceftriaxone be used to treat an infection caused by *Escherichia coli* that is susceptible to ceftriaxone but resistant to piperacillin-tazobactam?
6. The UL54 resistance mutations confers potential resistance to what antiviral(s)?

Clinical microbiology

1. Should human herpesvirus 6 cause false positive results on the BioFire FilmArray Meningitis/Encephalitis Panel?
2. Which serotypes does the *Legionella pneumophila* urinary antigen detect?
3. Should the two-tiered antibody test be performed for a patient presenting with erythema migrans?
4. What is the negative predictive value of a methicillin-resistant *Staphylococcus aureus* nasal swab PCR for pneumonia?
5. Does ampicillin susceptibility for *Enterococcus faecalis* confer susceptibility to meropenem?
6. Does *Candida albicans* growing on a fungal culture from a bronchoalveolar lavage require antifungal treatment?

**Supplementary Appendix 2**. Example of clinical infectious diseases pharmacotherapy questions and GPT-3.5 response based on question category.

***Drug Selection***

**
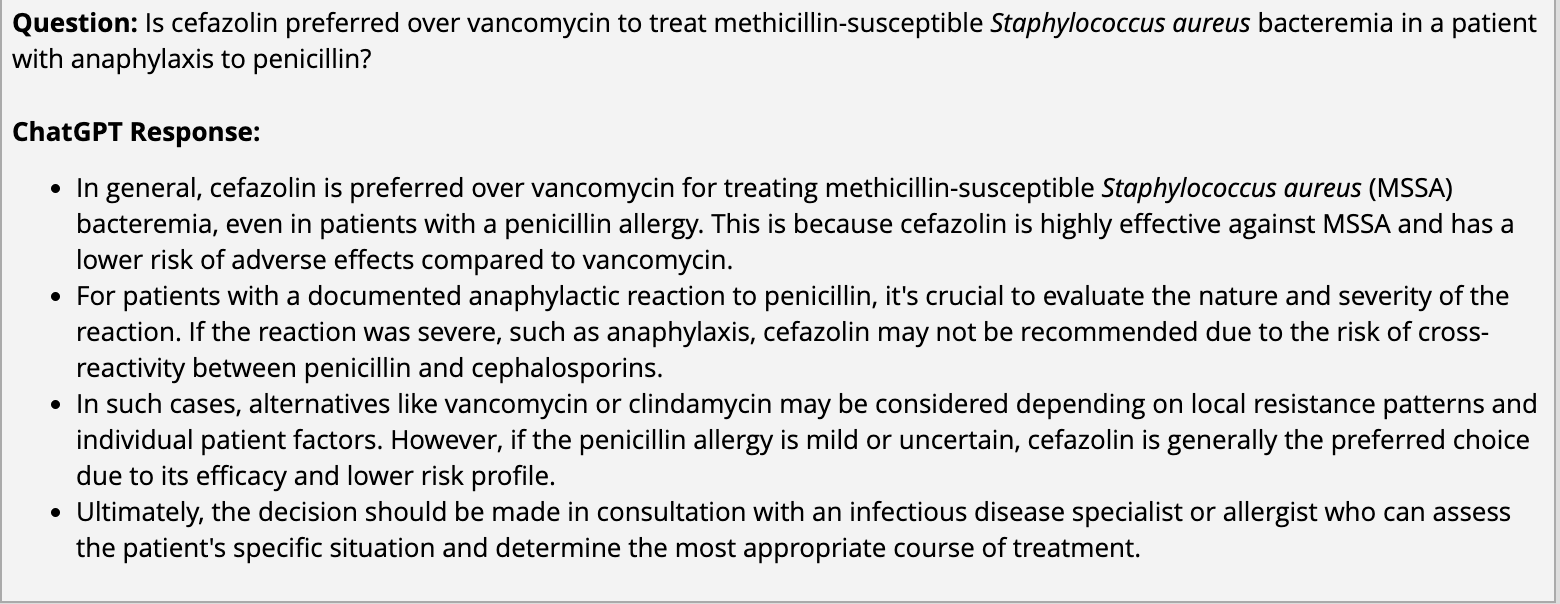
**

***Drug Dosing***

***
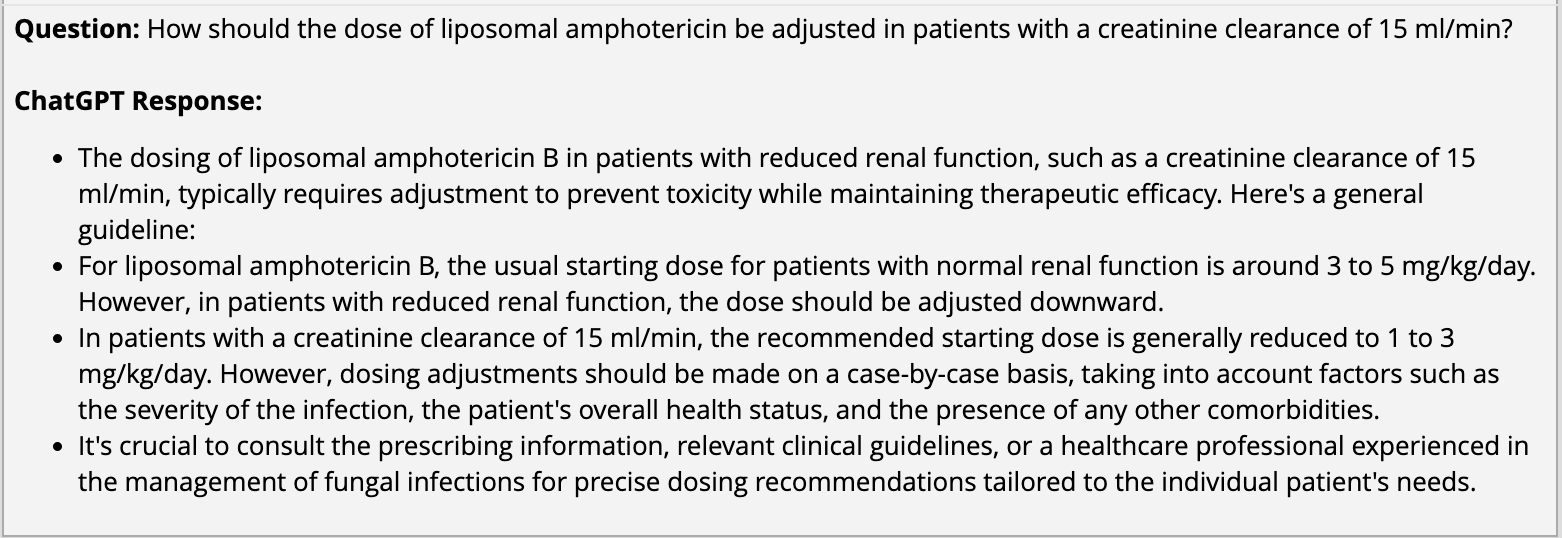
***

***Drug Interactions***

***
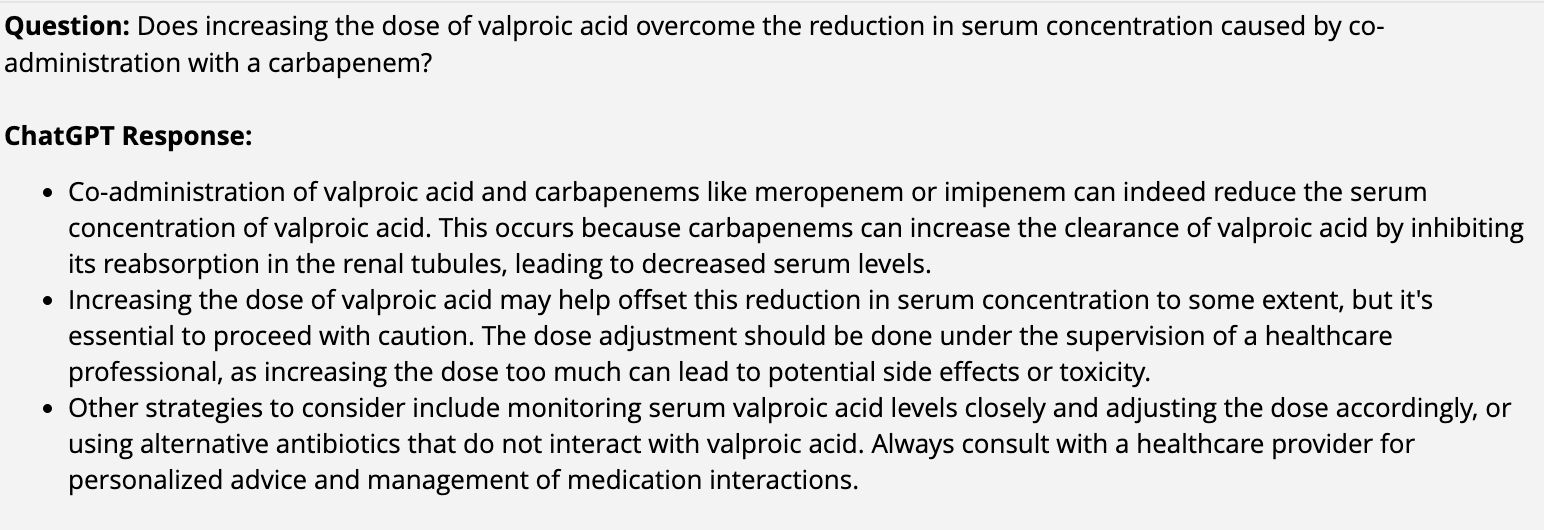
***

***Adverse Effects/Drug Monitoring***

***
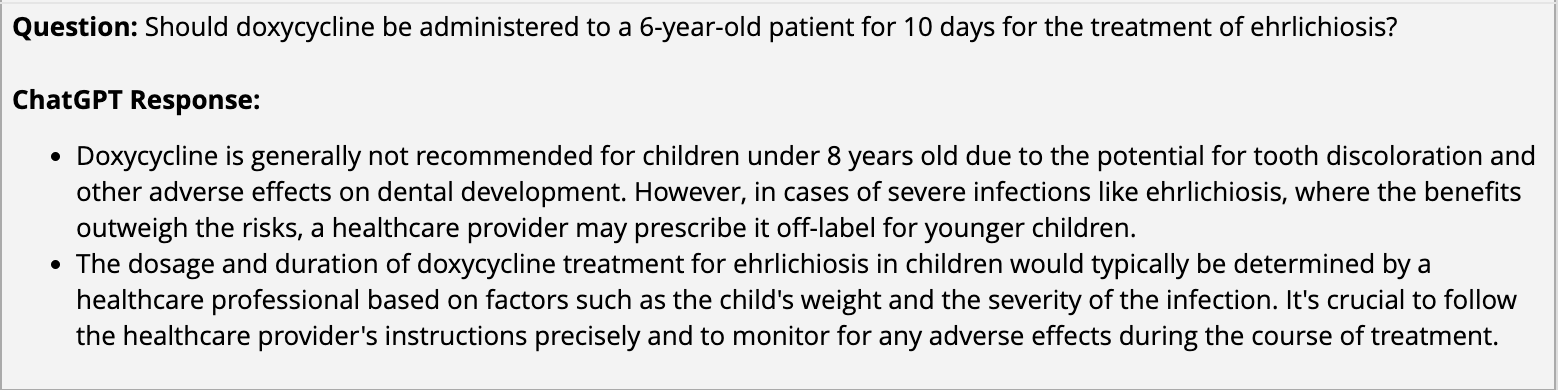
***

***Therapeutic Drug Monitoring***

***
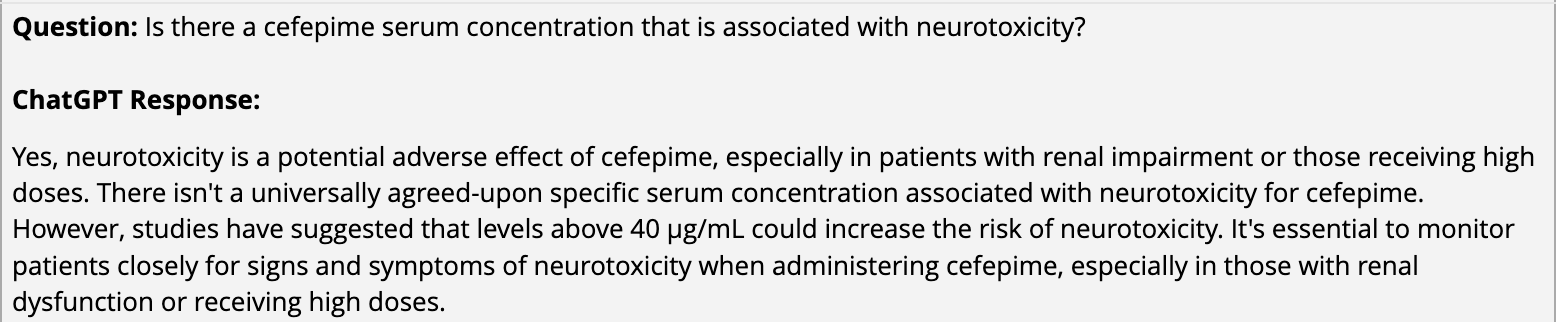
***

***Antimicrobial Prophylaxis***

***
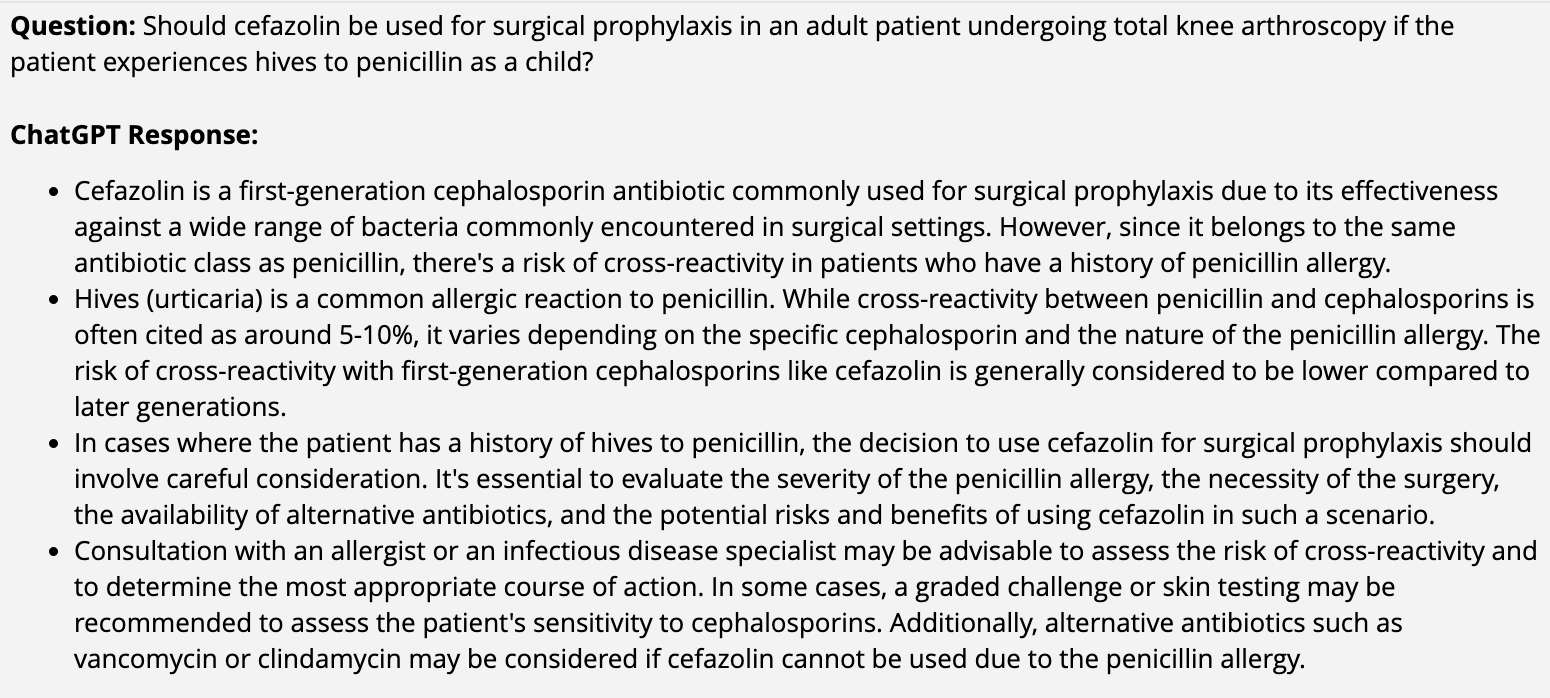
***

***Antimicrobial Resistance***

***
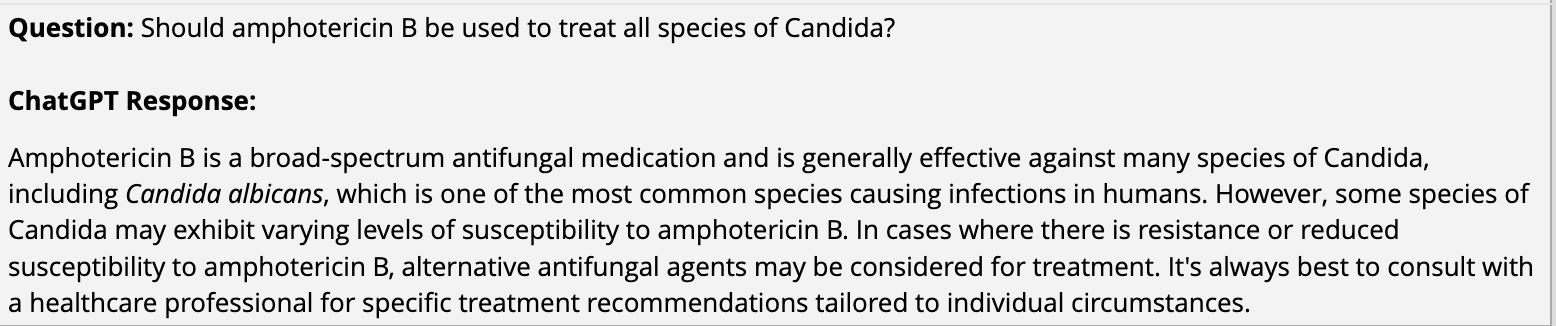
***

***Clinical Microbiology***

***
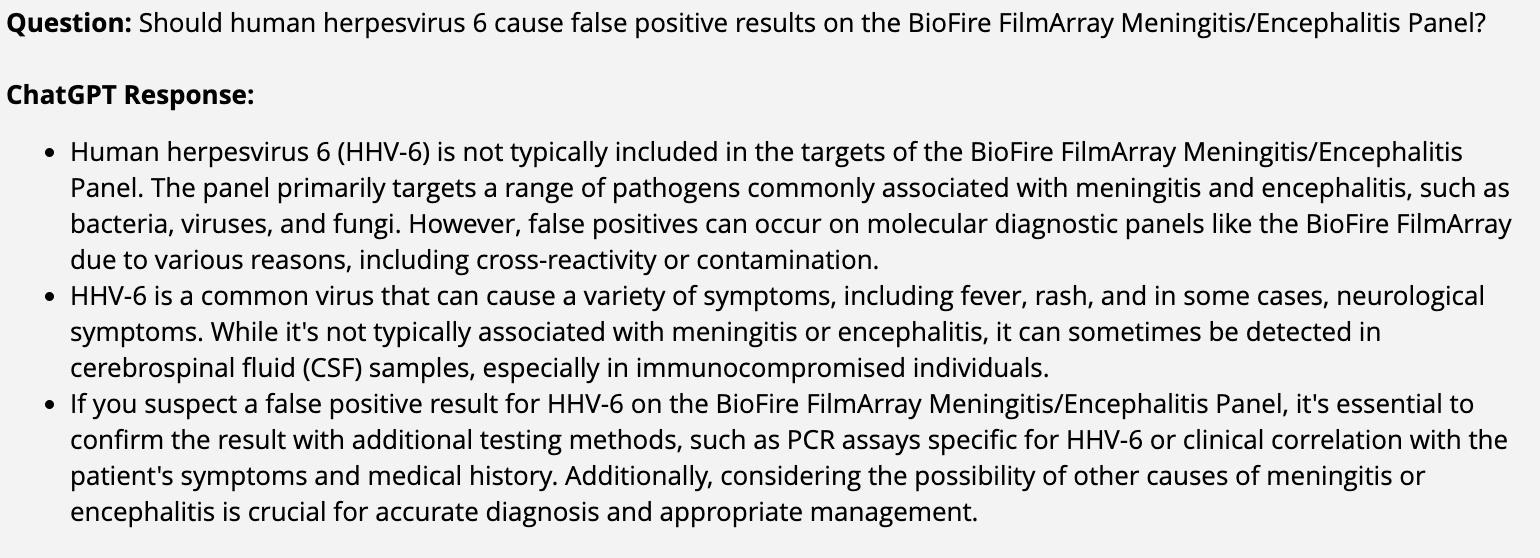
***

**Supplementary Appendix 3**. Example of clinical infectious diseases pharmacotherapy questions and GPT-3.5 response based on easy, medium, and hard ratings.

***‘Easy’ Questions***


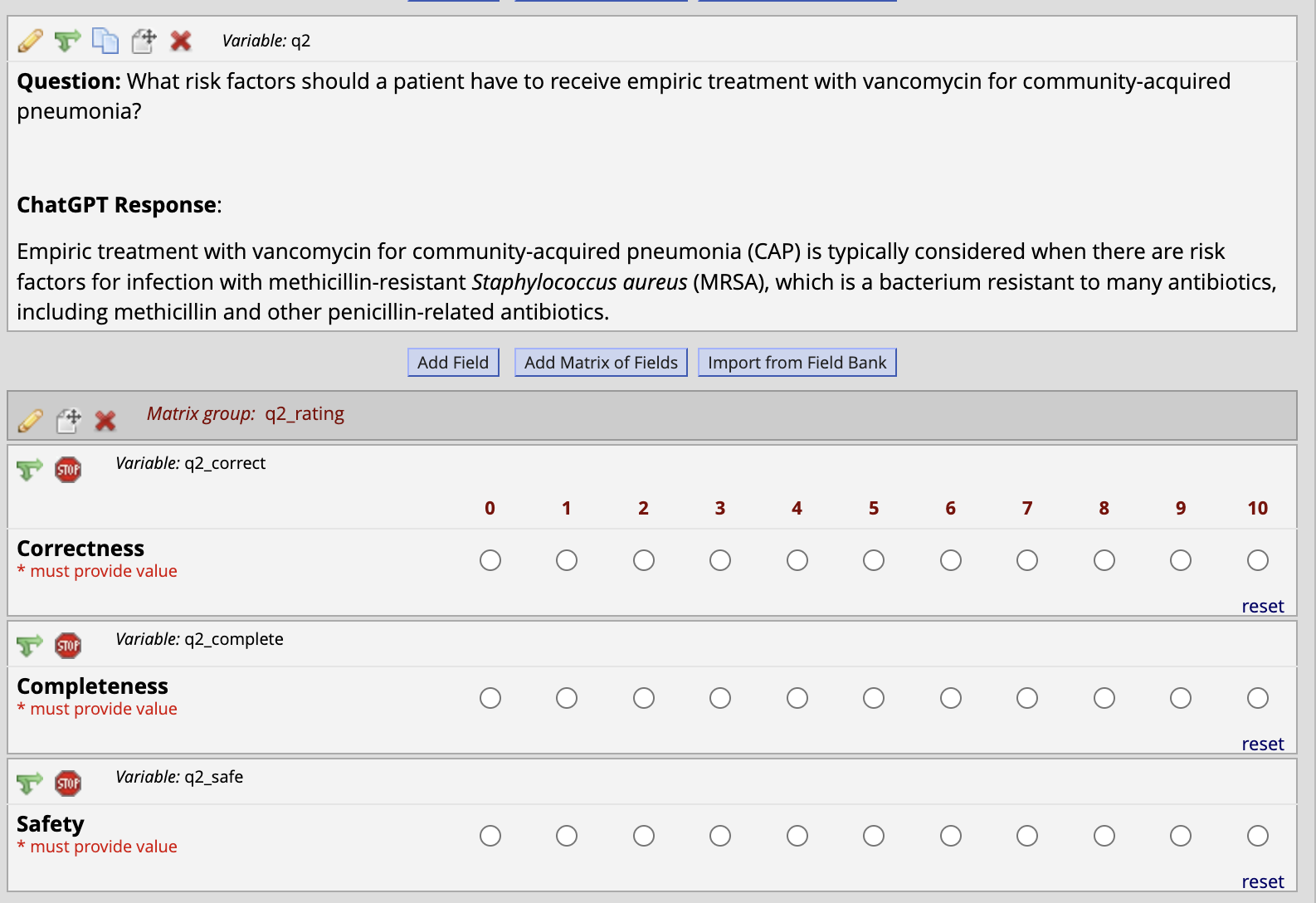


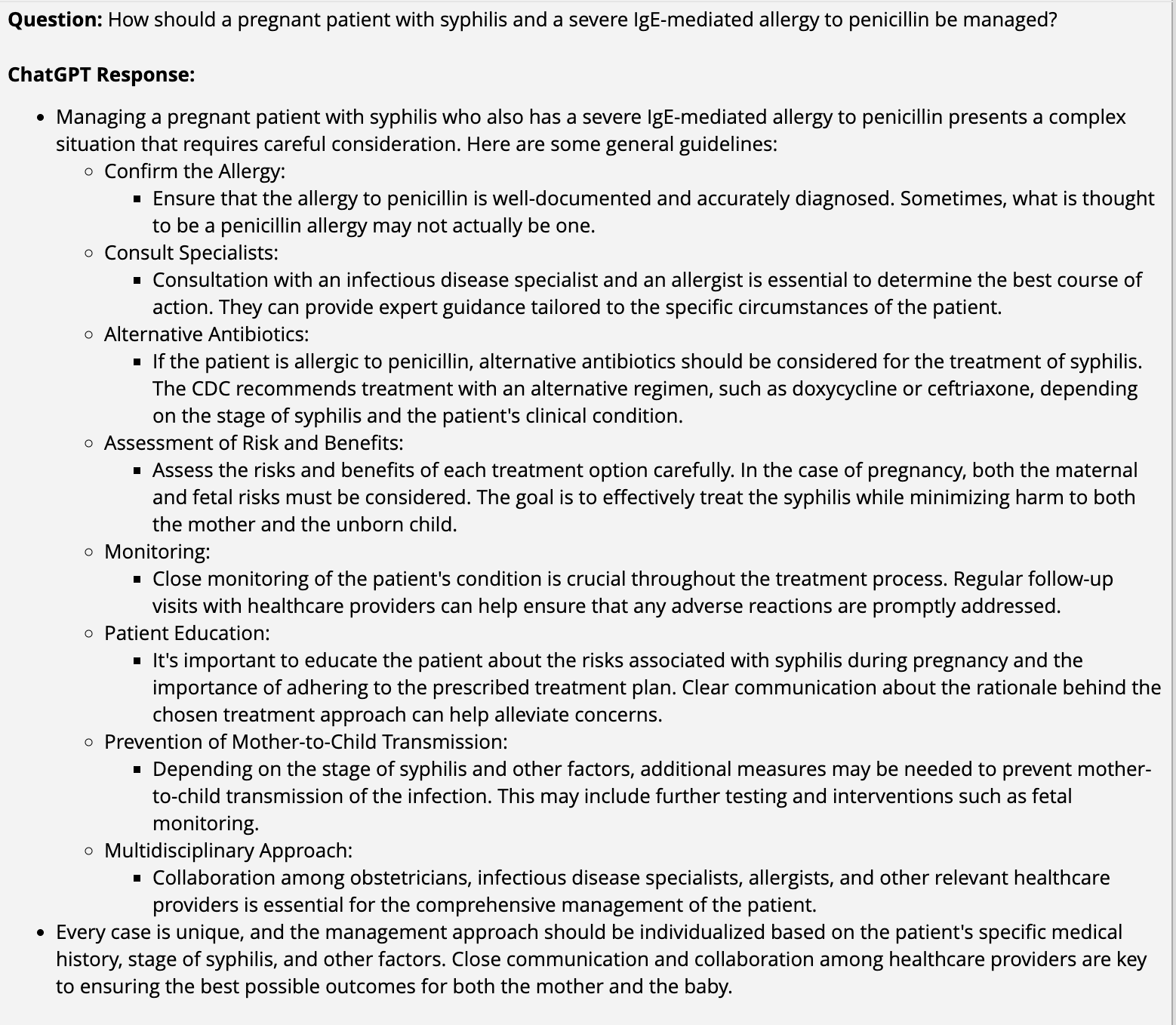


***‘Medium’ Questions***


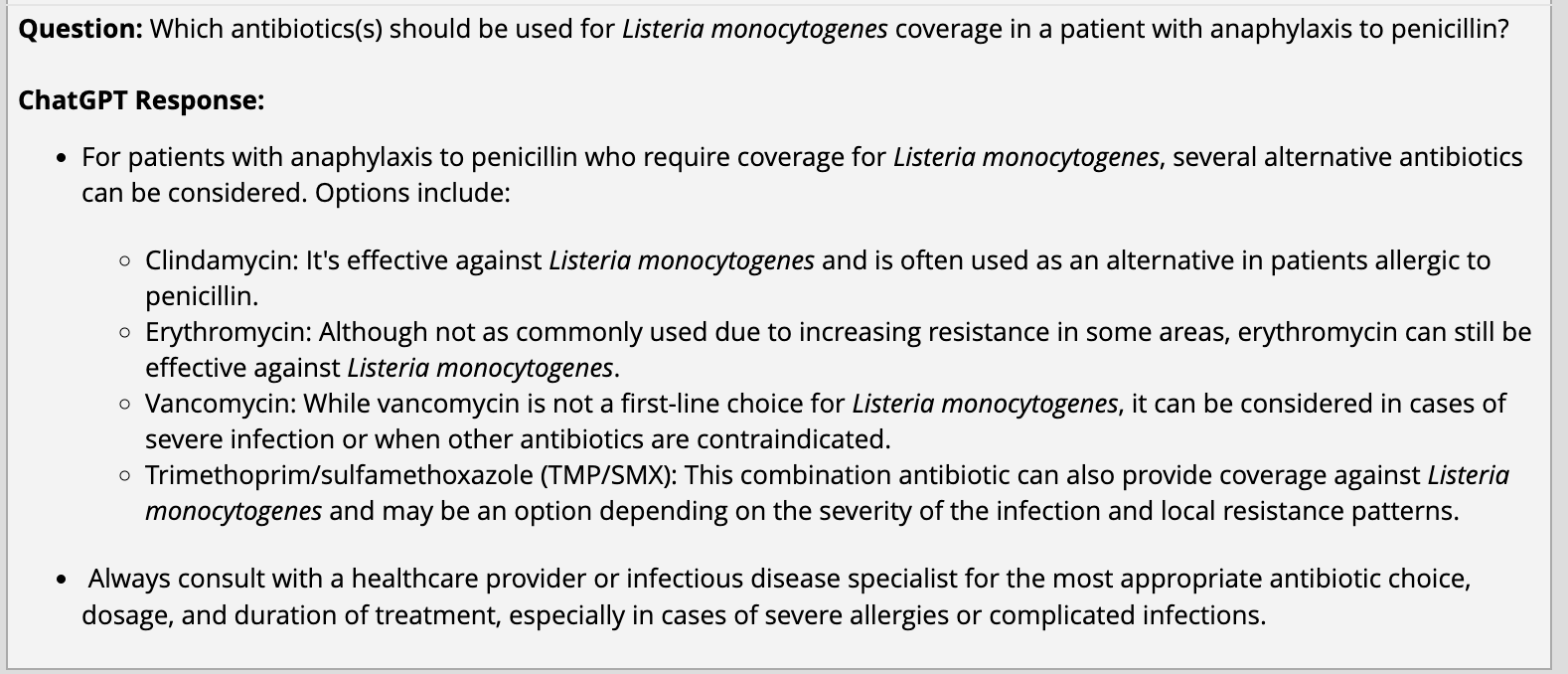


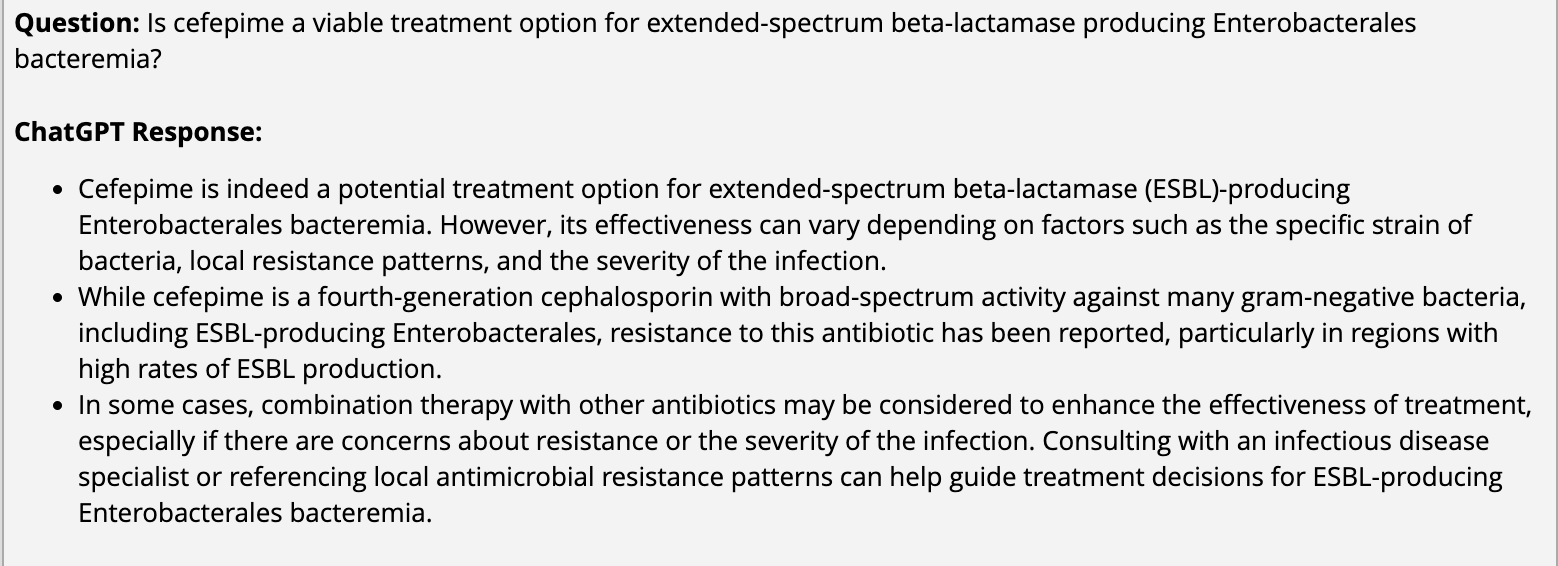


***‘Hard’ Questions***


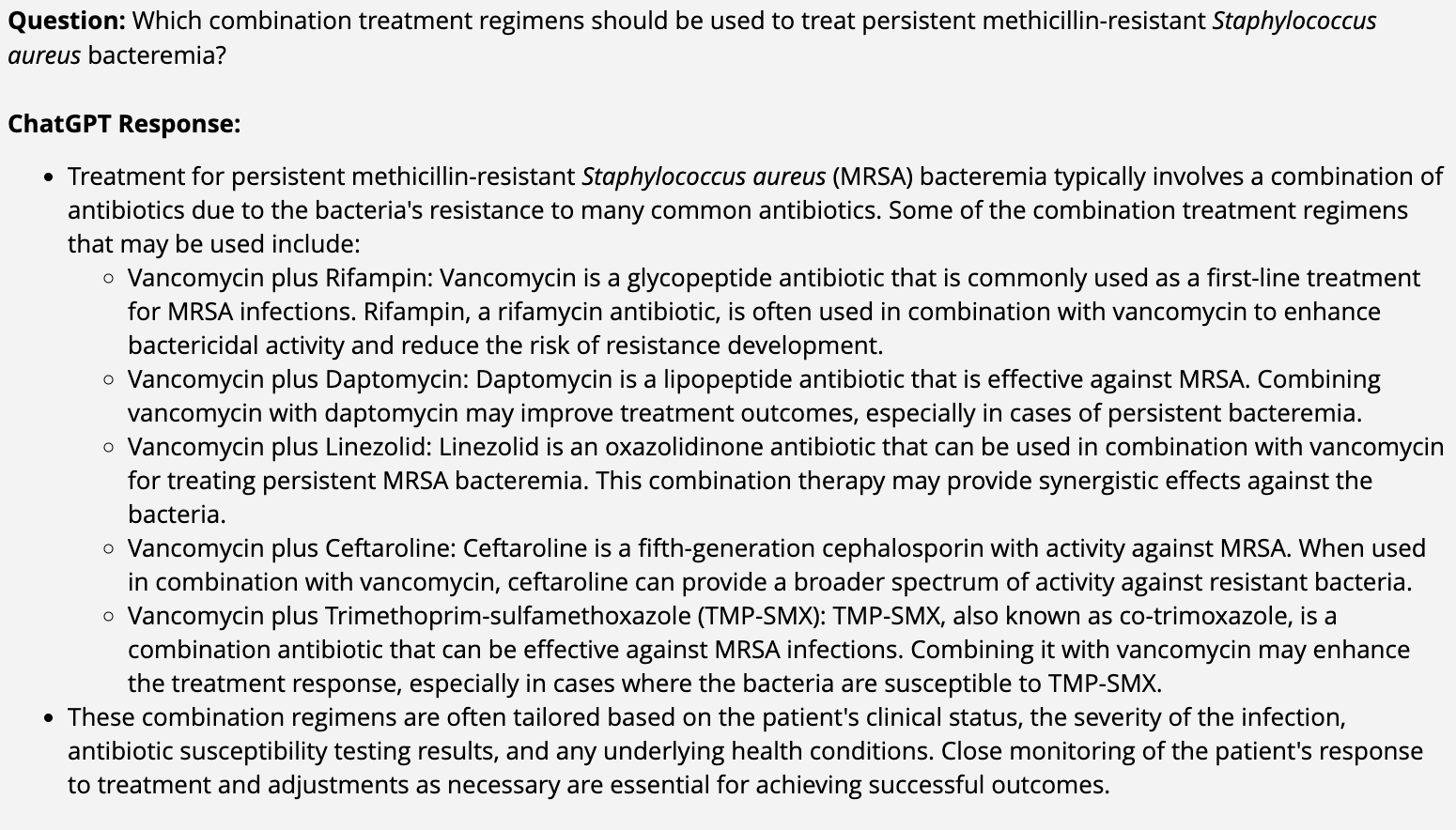


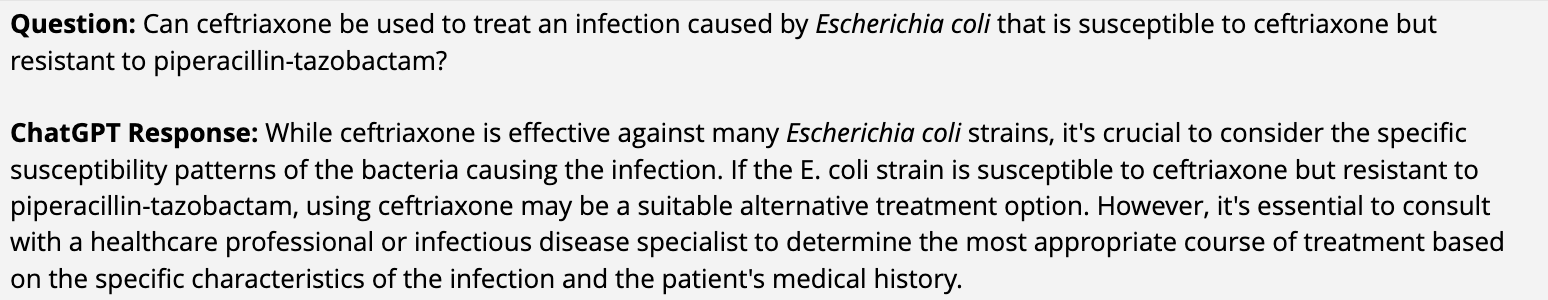


**Supplementary Table 1**. Demographics of the subject matter experts.

| **SME** | **Gender** | **Race/Ethnicity** | **Years of Practice** | **Degree(s)/**  **Certification(s)** | **Post-Graduate Training** | **Primary Clinical Practice Site** |
| --- | --- | --- | --- | --- | --- | --- |
| 1 | Female | White/European | 0-5 | PharmD  BCIDP | PGY-1  PGY-2 ID | Academic Medical Center |
| 2 | Male | White/European | 16-20 | PharmD  Masters  BCIDP | PGY-1  PGY-2 ID  ID Fellowship | Academic Medical Center |
| 3 | Male | White/European | >20 | PharmD  BCPS | PGY-1  PGY-2 ID | Academic Medical Center |
| 4 | Female | White/European | 6-10 | PharmD  BCIDP | PGY-1 | Community Teaching Hospital |
| 5 | Male | Middle Eastern/North African | 16-20 | PharmD  BCIDP | PGY-1 | Community Teaching Hospital |

Abbreviations: BCIDP, board-certified infectious diseases pharmacist; BCPS, board-certified pharmacotherapy specialist; ID, infectious diseases; PGY-1, post-graduate year-1 residency training; PGY-2, post-graduate year-2 residency training; SME, subject matter expert.
